# Open access to novel dual flow chamber technology for *in vitro *cell mechanotransduction, toxicity and pharamacokinetic studies

**DOI:** 10.1186/1475-925X-6-46

**Published:** 2007-12-04

**Authors:** Eric J Anderson, Melissa L Knothe Tate

**Affiliations:** 1 Department of Mechanical & Aerospace Engineering, Case Western Reserve University, Cleveland, OH, USA; 2 Department of Biomedical Engineering, Case Western Reserve University, Cleveland, OH, USA

## Abstract

**Background:**

A major stumbling block for researchers developing experimental models of mechanotransduction is the control of experimental variables, in particular the transmission of the mechanical forces at the cellular level. A previous evaluation of state of the art commercial perfusion chambers showed that flow regimes, applied to impart a defined mechanical stimulus to cells, are poorly controlled and that data from studies in which different chambers are utilized can not be compared, even if the target stress regimes are comparable.

**Methods:**

This study provides a novel chamber design to provide both physiologically-based flow regimes, improvements in control of experimental variables, as well as ease of use compared to commercial chambers. This novel design achieves controlled stresses through five gasket designs and both single- and dual-flow regimes.

**Results:**

The imparted shear stress within the gasket geometry is well controlled. Fifty percent of the entire area of the 10 × 21 mm universal gasket (Gasket I, designed to impart constant magnitude shear stresses in the center of the chamber where outcome measures are taken), is exposed to target stresses. In the 8 mm diameter circular area at the center of the chamber (where outcome measures are made), over 92% of the area is exposed to the target stress (± 2.5%). In addition, other gasket geometries provide specific gradients of stress that vary with distance from the chamber inlet. Bench-top testing of the novel chamber prototype shows improvements, in the ease of use as well as in performance, compared to the other commercial chambers. The design of the chamber eliminates flow deviations due to leakage and bubbles and allows actual flow profiles to better conform with those predicted in computational models.

**Conclusion:**

The novel flow chamber design provides predictable and well defined mechanical forces at the surface of a cell monolayer, showing improvement over previously tested commercial chambers. The predictability of the imparted stress improves both experiment repeatability as well as the accuracy of inter-study comparisons. Carefully controlling the stresses on cells is critical in effectively mimicking *in vivo *situations. Overall, the improved perfusion flow chamber provides the needed resolution, standardization and *in vitro *model analogous to *in vivo *conditions to make the step towards greater use in research and the opportunity to enter the diagnostic and therapeutic market.

## Background

Elucidation of cellular mechanisms of mechanotransduction is a critical step in uncovering physiologic mechanisms of tissue generation and repair. The biochemical and biophysical environment of virtually every cell in the body is affected by transduction of mechanical forces and chemical signals. Numerous research studies have used flow chambers to study the transduction of mechanical forces to cells, including those of the vascular endothelium [1], bone [2-7], renal proximal tubules [8], and other tissues [9-13]. However, a major stumbling block for researchers developing experimental models of mechanotransduction is the control of experimental variables, in particular the transmission of the mechanical forces at the cellular level.

The current state of the art comprises the parallel-plate flow chamber, where fluid perfuses across the specimen between critically spaced plates that make up the top and bottom of the chamber [2,3,14,15]. Variance in this basic design stems from the plate spacing as well as gasket geometry that constrains the fluid from inlet to outlet and controls the expansion and contraction of flow. Numerous redesigns and optimizations have been implemented in recent years to tailor parallel-plate chambers for specific cell experiments [16-19]. However, a recent study has shown that several commercial chambers do not perform well at imparting a known and controlled shear stress to a cell monolayer. This lack in performance complicates and compromises the validity of data comparison between studies [20].

Furthermore, it is not clear to how well perfusion chambers emulate aspects of physiologic flow regimes including spatiotemporal control of stress magnitudes and gradients. The common parallel-plate design found in many chambers incorporates a one-sided shear stress imparted to the cell monolayer. In typical experimental use, cells are seeded on a coverslip that is placed on the chamber floor; fluid moves over the apical surface of the cells, subjecting them to shear stress via fluid drag. Physiologically, cells may experience a more complex or multidimensional stress. For example, in the context of developing tissues (during development or engineering of tissues), cells experience multidimensional stresses [21-24]. In fully developed tissues, cells lining flow channels that themselves dilate and contract are exposed predominately to flow along the apical surface and strain on the basal surface. In contrast, cells within interstitial tissues may experience flow on apical and basal surfaces. Thus, cellular responses to flow regimes comprised of apical stimulation alone or basal stimulation alone may not accurately represent the physiologic environment within tissues. This provides impetus for the development of an experimental platform with the utility to "tune" experimentally applied stress regimes to better emulate the physiologic environment of a given cell type.

The purpose of this study was to identify and address weaknesses in current chamber designs by designing and testing a novel flow chamber that provides a more physiologic fluid environment for the study of cells *in vitro*. Hence, we created a modular perfusion flow chamber for transmission of fluid shear stress on either or both the apical and basal surfaces of cells. In addition, we developed and tested novel gasket geometries to improve upon current chamber designs and to elicit controlled stress regimes that mimic specific aspects of physiological flow on cells.

## Methods

In general, parallel plate flow chambers are designed to provide controlled flow of fluid across a cellular monolayer. Furthermore, flow profiles are generally controlled by the mass flow rate into the chamber and by the geometry of the channel through which the fluid flows. The geometry of the channel is typically defined by the surface of the substrate on which cells are seeded, bounded above by a compliant gasket and to the sides by the walls of the chamber and/or the gasket. We expand on this general design principle to create a chamber, and in effect a fluid geometry (via gasket design), that provides a physiologic and controlled mechanical stimulus to cells. We set two goals for the novel chamber, namely to construct a device that provides either single- or dual-flow regimes across the monolayer and to ensure controlled spatiotemporal distribution of shear stress on the cells through definition of appropriate gasket geometries. In addition, we placed particular attention on design for ease of use, as tests with commercial chambers had previously raised concerns with regard to leakage, bubble production, and lack of reproducibility of experimental conditions [20].

### Chamber design

**Figure 1:**
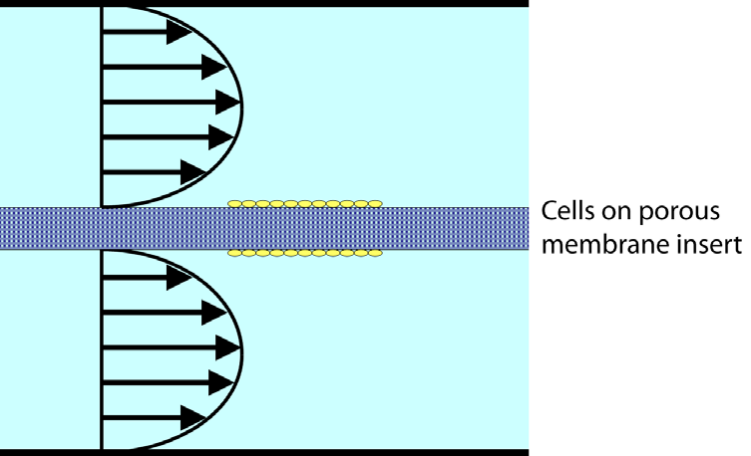
**Dual-flow profile schematic**. The general schematic for apical and basal flow used in the design of the novel flow chamber, where the cell monolayer is housed between the two fluid reservoirs on either a porous membrane or solid substrate.

To use the dual flow modality, cells seeded within and/or on one or both sides of a permeable membrane, or cells seeded in a scaffold disc, or cells *in situ *(in a slice of tissue), are interposed between the two gaskets and flow is applied. For example, cells are seeded on a porous membrane (0.2 micron Anapore membrane, Nunc International, Denmark) that is interposed between two gaskets. The cell monolayer is interposed between two identical fluid layers, each with an independent inlet and outlet (Figure 1). The dual configuration provides relatively even flow across both surfaces of the monolayer, where the thin membrane (thickness is chosen by the user) between the regimes suspends the specimen in a physiologic manner. However, as mentioned previously, the membrane can be interchanged with a variety of other substrates, depending on the specific needs of the study, *e.g*. a layer in which cells are seeded or embedded can be placed between the gaskets or a slice of tissue with cells *in situ *can also be interposed between the gaskets. Furthermore, single-flow can also be achieved with a solid coverslip in place of the membrane.

**Figure 2:**
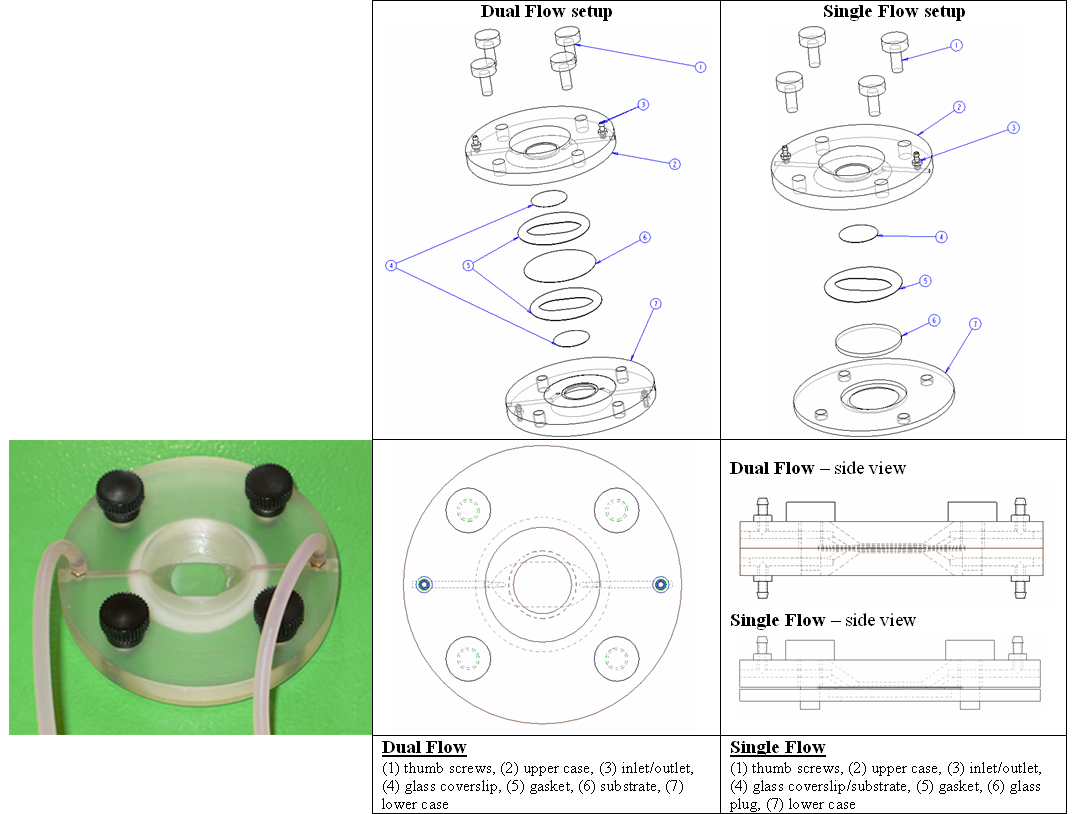
**Novel flow chamber design**. Flow geometry is dictated by the rubber gasket(s), where two polycarbonate cases are used to deliver flow as well as to compress and seal the fluid space. Nylatron thumb screws are used to tighten the chamber, and circular glass coverslips are used to seal flow and provide microscope visualization. Flow is administered through inlet and outlet tubing connected to the barbed inlets/outlets, placed orthogonal to the chamber surface for ease of use with the microscope stage. Left: fabricated flow chamber in use for endothelial studies. A mouse embryonic stem cell line E14Tg2a is pre-differentiated on cover-slips coated with 0.1% gelatin, and then exposed to shear stress of 1.5 – 5 dyn/cm^2 ^to induce endothelial differentiation. Typical experiments are carried out for 1–2 days (courtesy of Professor Horst von Recum). Right: technical drawings of design in dual and single mode.

Following the general flow design, the chamber itself consists of an upper and lower reservoir that is dictated by compressing rubber gaskets between two polycarbonate cases (Figure 2). The solid cases are fabricated such that the inlet and outlet channels are encased within the polycarbonate, in which the flow descends from the inlet channel, into the upper reservoir (formed by the geometry of the rubber gasket), and then exits via the polycarbonate outlet channel. Aside from the inlet/outlet channels, the flow regime, from the perspective of the cellular monolayer, is completely governed by the rubber gasket geometry as well as a glass coverslip above the monolayer which enables microscope visualization into the chamber. It should be noted that single-flow is achieved by the replacing the membrane with a glass coverslip, or by using a glass plug which completely "fills" the lower reservoir, decreasing the overall thickness of the chamber for ease of use when single-flow is desired.

### Gasket design

**Figure 3:**
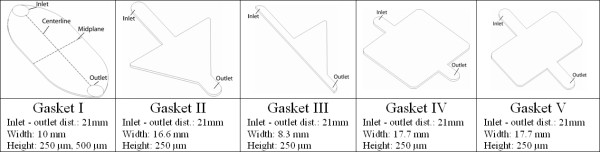
**Gasket designs**. (I) Designed to impart constant shear stress to the cell monolayer; (II) symmetric expansion, linear contraction zone for specific stress gradient; (III) asymmetric expansion, linear contraction zone for specific stress gradient; (IV) planar jet geometry for specific stress gradient; (V) shortened planar jet geometry.

Five different gasket geometries are created (Figure 3) to provide a variety of controlled stress fields to the cell monolayer, depending on the desired application,. Each gasket is compatible with the general parallel plate configuration; geometric differences control the specific spatial variation of shear stress imparted to the cell. In gasket I the geometry is designed to provide a constant shear stress to the majority of the cellular monolayer. In this case, the geometry contains zones of widening and tapering geometry that surround the parallel walls near the chamber midplane. In gasket II and III, the gasket geometry contains symmetric and asymmetric wide flow zones, whose walls taper immediately and linearly as they approach the outlet. Thus, the geometry provides specific gradients of stress to a monolayer. Finally, the last two gasket designs are designed to provide planar jet like flow, where zones with geometries that widen and taper abruptly define two different flow geometry lengths. Again, the geometry is created such that specific gradients of stress would be imparted to the cells, as opposed to the constant stress environment of gasket I.

### Computational fluid dynamics

Computational models are created for each design to predict the fluid environment and imparted shear stress in each gasket. The inlet and outlet geometries do not change within the polycarbonate sections of the chamber. Hence, it is assumed that the entering and exiting flow are identical for each case and only the geometry within the rubber gaskets is modelled. Furthermore, the chamber design can accommodate a variety of gasket thicknesses. For the computational fluid dynamics (CFD) study, all gaskets are created with a thickness of 250 μm for comparison with previous predictions for commercial chambers [20]. In addition, gasket I is modelled for both 250 μm and 500 μm thicknesses to demonstrate flow variation attributable to gasket thickness. Gasket I is also modelled for single- and dual-flow scenarios, where the membrane is given a porosity of 50% and permeability of 10^-16 ^m^2^. All other gaskets are modelled in single-flow mode. Inter-geometric variations in dual flow mode can be inferred from gasket I simulation results. In all cases, the cells are not included in the gasket fluid geometry; the effect of flow on cells has been reported previously [20]. For each flow model (gaskets I-V), the number of volumes were 89760, 117130, 117130, 179380, and 189180, respectively. The mesh density was increased until grid independence was achieved for each gasket (average finite volume = 10^-13 ^m^3^).

The fluid environment is simulated for each model using a computational solver (CFD-ACE software package, Huntsville, AL) that calculates the velocity, pressure, and shear stress distribution. Flow is induced via a pressure gradient for each model to achieve a target wall shear stress at the exposed surface of the cell (at the interface with the flow channel's lower surface) is 1 dyn/cm^2 ^[23-25,7]. Also, no-slip conditions are enforced on the walls of each gasket. For all models, the continuity equation (1) and Navier-Stokes equations (2) are solved using a 2^nd ^order upwind-discretization scheme in three dimensions, and the wall shear stress is calculated from the wall strain rate (3).

∇·*u *= 0

*p*(*u*·∇*u*) = -∇*p *+ *μ*∇^2^*u*

τwall=μγ˙
 MathType@MTEF@5@5@+=feaafiart1ev1aaatCvAUfKttLearuWrP9MDH5MBPbIqV92AaeXatLxBI9gBaebbnrfifHhDYfgasaacPC6xNi=xI8qiVKYPFjYdHaVhbbf9v8qqaqFr0xc9vqFj0dXdbba91qpepeI8k8fiI+fsY=rqGqVepae9pg0db9vqaiVgFr0xfr=xfr=xc9adbaqaaeGacaGaaiaabeqaaeqabiWaaaGcbaacciGae8hXdq3aaSbaaSqaaiabdEha3jabdggaHjabdYgaSjabdYgaSbqabaGccqGH9aqpcqWF8oqBcuWFZoWzgaGaaaaa@3809@

where *u *is the velocity vector, *ρ *is the fluid density, *p *is the pressure, *μ *is the fluid viscosity, *τ *is shear stress, and γ˙
 MathType@MTEF@5@5@+=feaafiart1ev1aaatCvAUfKttLearuWrP9MDH5MBPbIqV92AaeXatLxBI9gBaebbnrfifHhDYfgasaacPC6xNi=xH8viVGI8Gi=hEeeu0xXdbba9frFj0xb9qqpG0dXdb9aspeI8k8fiI+fsY=rqGqVepae9pg0db9vqaiVgFr0xfr=xfr=xc9adbaqaaeGacaGaaiaabeqaaeqabiWaaaGcbaacciGaf83SdCMbaiaaaaa@2D8A@ is the strain rate. In all cases, the fluid is assumed to be similar to water and given a viscosity = 0.001 kg/ms and density = 1000 kg/m^3^. Flow is simulated for steady conditions with a local convergence criterion of 0.0001, where conservation of mass is used to validate each solution. It is important to note that, while simulations are calculated to achieve a target shear stress of 1 dyn/cm^2^, the results of these simulations can be scaled up for flow regimes in the laminar range (appropriate assumptions for flow regimes up to and beyond 20 dyn/cm^2^, i.e. regimes commonly applied for mechanotransduction research).

### Chamber testing

In order to evaluate the chamber design in a realistic laboratory setting, bench-top testing is performed to determine ease of use and performance. During testing, both strengths and weaknesses are noted in comparison to the previously evaluated commercial chambers [20]. Ease of use testing consists of assembling the chamber for fluid/cell experiments, with emphasis placed on the choice of the materials and geometry for each design and how the chambers interface with standard laboratory equipment used in flow/cell studies (i.e. tubing, microscope, syringe pump, etc.). However, performance testing consists of actual flow through the assembled chamber, where problems associated with leaking, bubbling, visualization, etc. are the main areas of interest. In both modes, the novel design is compared with existing designs, providing a basis to evaluate improvement over the current state-of-the-art.

## Results

### Gasket I

**Figure 4:**
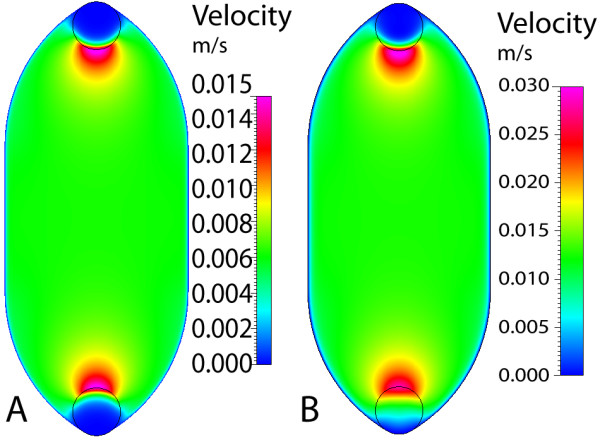
**Gasket I velocity in single-flow**. Maximum axial velocity plane, center of gasket height, for gasket thickness of (A) 250 μm and (B) 500 μm.

**Figure 5:**
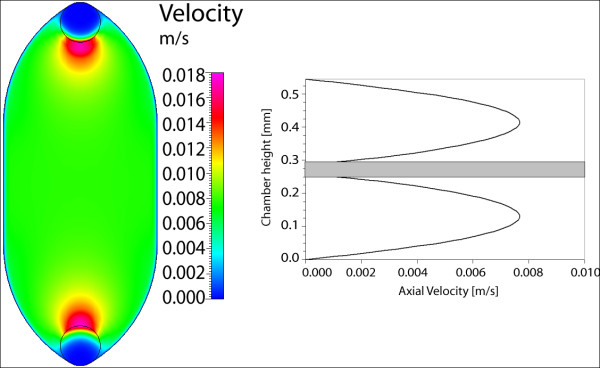
**Gasket I velocity in dual-flow**. (A) Maximum axial velocity plane in dual-flow setup, upper gasket; (B) Velocity profiles for upper and lower gaskets in dual-flow, at intersection of centerline and midplane.

**Figure 6:**
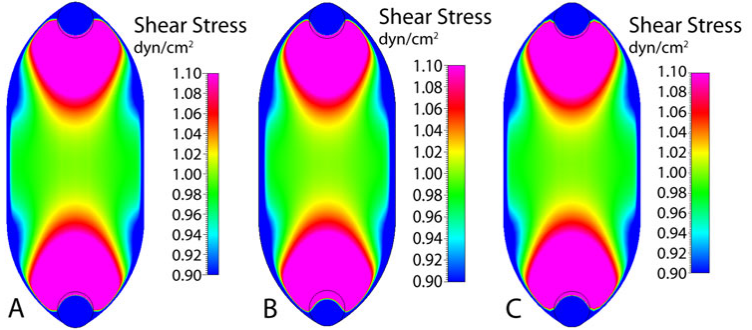
**Gasket I shear stress**. Wall shear stress on bottom wall of chamber, at location of cell monolayer, for (A) single-flow 250 μm gasket, (B) single-flow 500 μm gasket, and (C) dual-flow 250 μm gasket.

**Figure 7:**
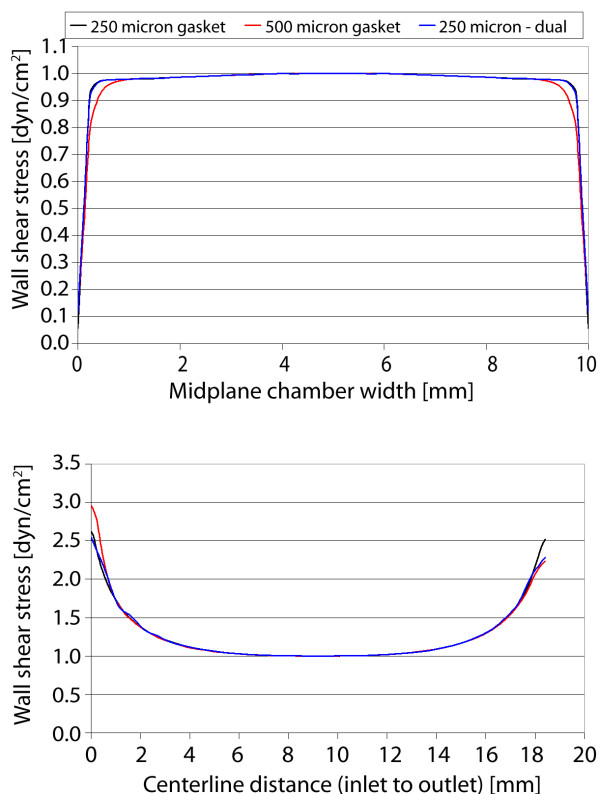
**Gasket I shear stress plots**. Wall shear stress plotted along chamber midplane (top) and centerline (bottom) for the three cases.

**Figure 8:**
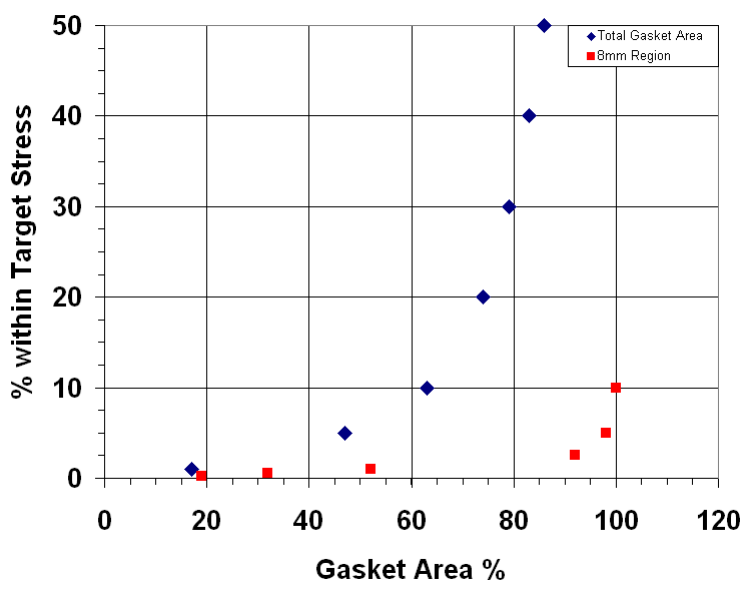
**Gasket I area and shear stress range**. The percentage of area that is within the specified range of the target shear stress for (a) entire gasket area and (b) an 8 mm region of interest.

For the first gasket geometry, the maximum velocity and wall shear stress are calculated for three cases: (a) 250 μm gasket thickness, (b) 500 μm thickness, and (c) dual-flow 250 μm thickness (each layer). The maximum velocity in each case is highest near the inlet/outlet, with a nearly constant magnitude throughout the rest of the geometry (displayed within the plane) (Figure 4). As the thickness or gasket height is doubled from 250 to 500 μm, the maximum velocity (at the center height) increases proportionally. However, when the 250 μm gasket is used in dual-flow mode, the maximum velocity increases by 20% as compared to the single-flow setup (Figure 5). In all cases the calculated velocity is designed to impart a target wall shear stress of 1 dyn/cm^2 ^on the chamber bottom (at the cell monolayer location). Here, the shear stress distributions are similar for the three cases (Figure 6). As seen in midplane and centerline plots, the shear stress achieves the target magnitude near the middle-third of the geometry. The imparted stress is nearly constant over the midplane, where over 95% (9.5 mm) achieves the target stress within 5% (Figure 7). In addition, nearly 45% (8.2 mm) of the centerline (inlet to outlet distance) attains the target stress within 5%, thus 47% of the total gasket area experiences a shear stress within 5% of the target value (Figure 8). If an 8 mm region of interest is considered, centered on the chamber floor, in order to compare to commercial chambers, it is found that 60% of the region is within 1% of the target stress, over 92% is within 2.5% of the target, and the entire region is within 10% of the target stress. As the allowable range of shear stress (within the target) increases, the area of the gasket that feels this imparted stress increases as well.

### Gasket II

**Figure 9:**
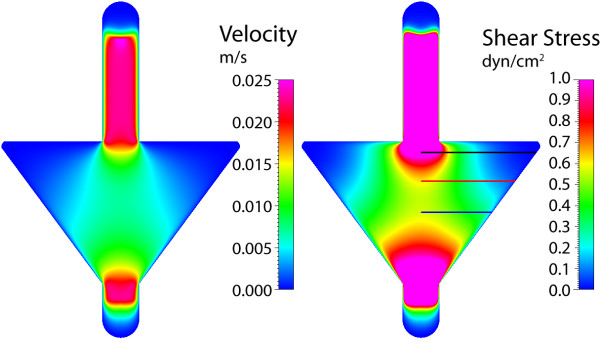
**Gasket II velocity and shear stress**. Maximum axial velocity plane (left), and wall shear stress on chamber bottom (right) with three sampling locations for shear stress profile.

**Figure 10:**
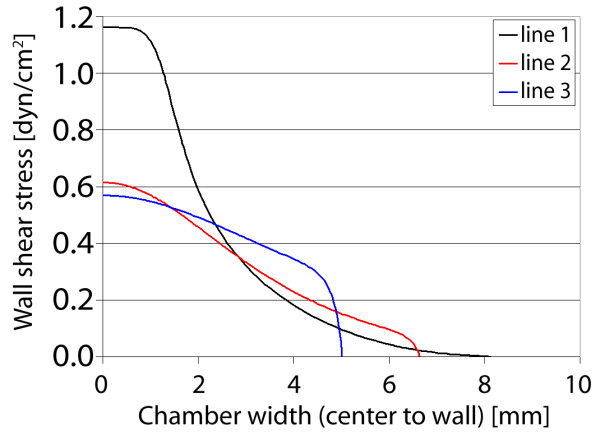
**Gasket II shear stress plots**. Wall shear stress for the three sampling locations, plotted from the chamber centerline to the wall. High gradients of shear stress are found near the entrance to the expansion zone (line 1), where the imparted stress smooths with increasing distance from the entrance (near midplane).

For the second gasket, the instantaneous widening and subsequent gradual tapering of the walls yield a unique velocity and stress distribution (Figure 9). As flow enters the wide zone, the velocity dissipates immediately, similar to planar jet flow. However, due to the subsequent tapering walls of the gasket, the maximum velocity is almost sustained along the centerline (within the expansion zone), approaching zero in the outer regions of the gasket. This distribution corresponds to defined gradients of shear stress imparted to the chamber floor, again for a target of 1 dyn/cm^2^. The shear stress reaches a maximum near the entrance to the widening and tapering zones. Between the zones of fluid "expansion" and "contraction" (corresponding to widening and tapering of the flow channel walls), varying gradients of stress are found within the gasket. Focusing on three different locations within the gasket, initially a sharp gradient in stress is found between the gasket center and walls (Figure 10). However, moving away from the entrance to the "fluid expansion" zone, the shear stress decreases at the center and increases near the walls, essentially smoothing the stress distribution. Thus, the shear stress and local variation in stress are dependent on their location along the centerline.

### Gasket III

**Figure 11:**
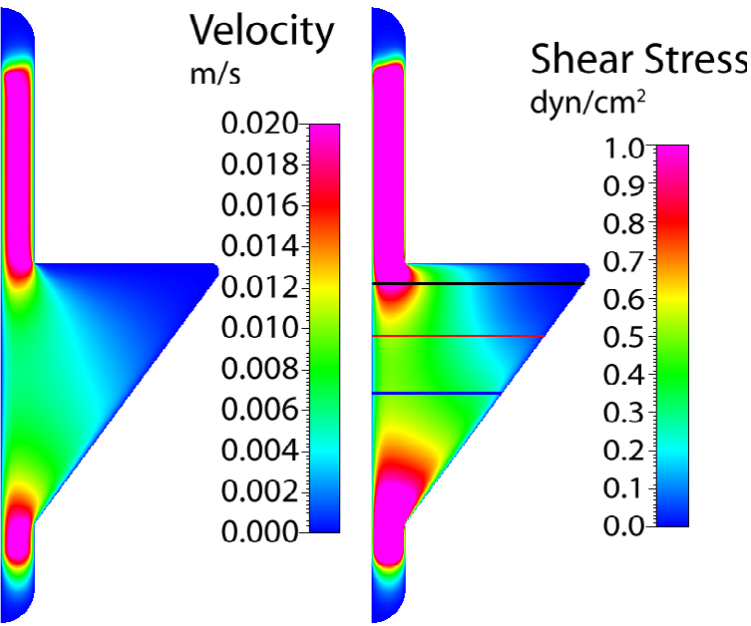
**Gasket III velocity and shear stress**. Maximum axial velocity plane (left), and wall shear stress on chamber bottom (right) with three sampling locations for shear stress profile.

**Figure 12:**
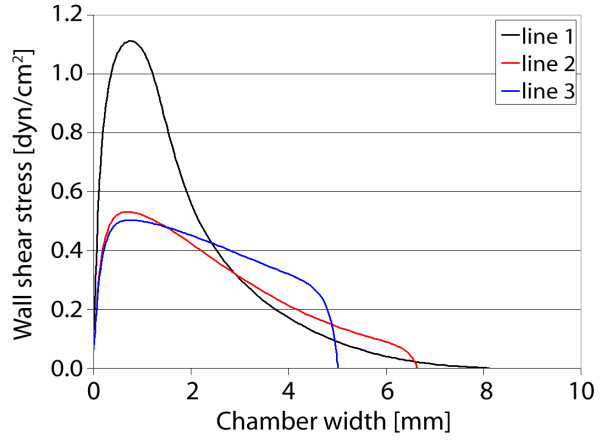
**Gasket III shear stress plots**. Wall shear stress for the three sampling locations, plotted from the chamber centerline to the wall. Similar to Gasket II, however sharp gradients of stress are found near the centerline or left wall of the gasket.

Gasket III is the asymmetric version of the previous gasket design, where only one half of the fluid expansion zone (where the channel area widens) is created. Here, the velocity again appears similar to planar jet flow due to the widening geometry. However due to the asymmetry and the no-slip condition, the velocity decreases sharply as it approaches the "centerline" wall (Figure 11). Again, this distribution causes defined gradients in shear stress on the chamber floor, where the maximum stress is found 0.75 mm from the "centerline" wall (Figure 12). The largest gradient is stress is found near the entrance to the widening channel, where the imparted stress is smoothed with increasing distance from the entrance. However in all cases, a sharp gradient of shear stress is found near the "centerline" wall, providing unique and defined stress distributions within the gasket.

### Gasket IV

**Figure 13:**
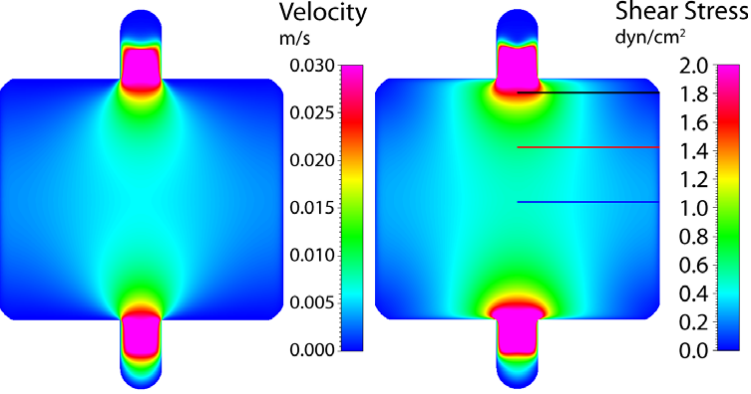
**Gasket IV velocity and shear stress**. Maximum axial velocity plane (left), and wall shear stress on chamber bottom (right) with three sampling locations for shear stress profile.

**Figure 14:**
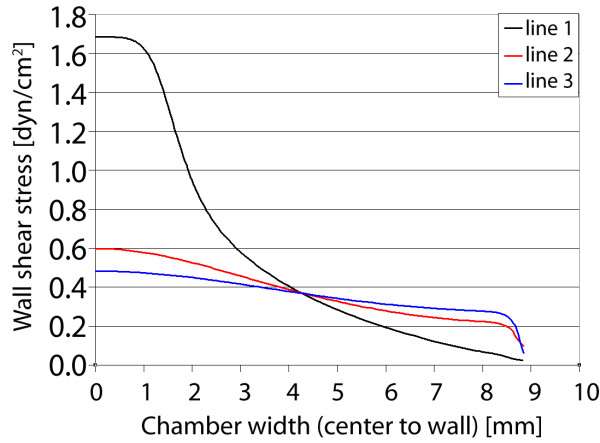
**Gasket IV shear stress plots**. Wall shear stress for the three sampling locations, plotted from the chamber centerline to the wall. High gradients of shear stress are found near the entrance to the expansion zone (line 1), with nearly constant profiles near the midplane (lines 2, 3).

The fourth gasket imposes the most abrupt widening and tapering geometry, where the resulting fluid regime parallels planar jet flow (Figure 13). The maximum velocity is found in the smaller sections of the gasket, near the inlet and outlet, where the magnitude dissipates within the widened flow area. Here, flow near the walls of the widened area has a velocity of nearly zero along the entire length of gasket. This profile causes an increased shear stress near the gasket centerline, with values decreasing with increasing distance from the center. At a location near the entrance to the widened area, the shear stress is nearly constant near the centerline, where a dramatic gradient is found roughly 1 mm from the center (Figure 14). As flow moves away from the entrance, the gradient in wall shear stress (from the center to walls) is decreased and a nearly constant stress is found at the midplane. This gasket geometry induces the largest change in stress between the widening entrance and the midplane, again providing unique and defined stress distributions.

### Gasket V

**Figure 15:**
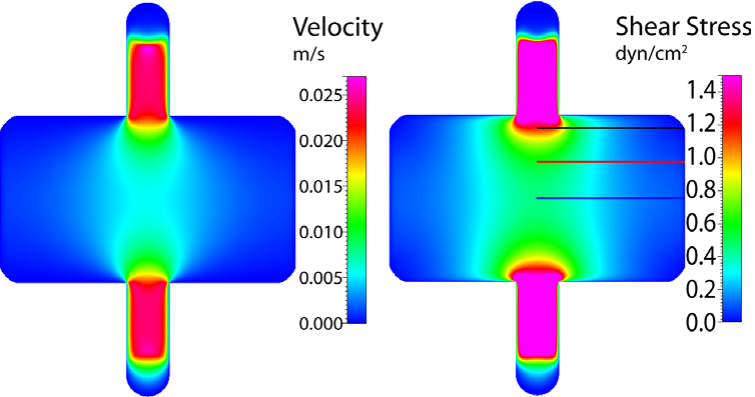
**Gasket V velocity and shear stress**. Maximum axial velocity plane (left), and wall shear stress on chamber bottom (right) with three sampling locations for shear stress profile.

**Figure 16:**
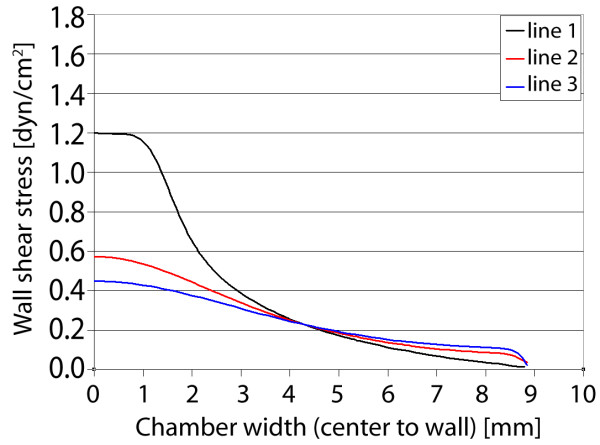
**Gasket V shear stress plots**. The magnitudes in stress are decreased from Gasket IV, where the midplane stress (line 3) is more variable as well.

Finally, Gasket V is a variation of the planar jet geometry of the previous gasket, where the length of the widened area is decreased. As a result, the required velocity decreases from that of Gasket IV in the inlet/outlet channels and near the transition zones to the widened channel area (Figure 15). The subsequent tapering of the area causes the change in shear stress between the entrance to the widened geometry and the midplane to be less dramatic than the previous geometry (Figure 16). The maximum shear stress at line 1 is again found at the centerline, however for a 33% reduction in the length of the expanded area (from Gasket IV), the maximum shear stress at this location is reduced by 29%. In addition, the centerline shear stress at lines 2 and 3 are similar to Gasket IV, however the decrease in stress near the gasket walls is greater for this geometry. Thus, the gradient of shear stress at the midplane is greater from the centerline to the wall due to tapering of the flow area.

### Chamber testing

Bench-top testing of the novel chamber prototype shows improvements, in the ease of use as well as in performance, compared to other commercial chambers (Table 1). The chamber is relatively easy to assemble, using a gasket compression design with four thumb screws, where silicon grease is used to ensure proper sealing and eliminate leakage found in other chambers. However, in conditions where sterility is crucial, assembly is more difficult, and the novel prototype requires comparable assembly times to the commercial chambers. In performance testing, the novel prototype reduces leakage and bubbling, up to flow rate of 60 mL/min which is a rate 10 times higher than that at which the commercial chambers show leakage and bubbling. Overall, the strength of the novel prototype chamber lies in its compression design, which eliminates flow deviations (leakage, bubbles) and allows actual flow profiles to better conform with those predicted computationally.

**Table 1:** Bench-top testing. Bench-top testing comparison for ease of use and performance between commercial and proposed chamber designs.

**Chamber type**	Oligene FCS	Bioptechs FCS2	Warner RC-30	ProFlow™
**Ease of Use**	• Straightforward assembly • Retaining ring difficult to position • Persistent leakage • Difficult to envision in use in sterile environment • Good for optical imaging • Convenient connection to syringe pump	• Difficult assembly • Stage adaptor fit both Zeiss and Leica scopes with ease • Many parts, "over engineered" • Tubing easily connected to syringe pump • Bottom coverslip extremely fragile	• Difficult assembly • Polymer gasket difficult to manage • Chamber fits together well • Difficult to seal chamber → leakage • Tubing poorly sized and too stiff	• Relatively easy to assemble • Vacuum grease needed to seal well but grease is never in contact with cells • Ease of use diminishes under sterile conditions
**Performance**	• Issues with sealing • 0.25–2 ml/min flow without leaking • Leakage at higher flow rates • Leakage between inlet and chamber body	• Major leakage unless extra spacer used • Needed much silicon grease to insure sealing, but then no leakage up to >36 ml/min • Numerous & persistent air bubbles and pockets formed inside chamber	• Major leakage problems • Max flow ~1–12 ml/min • No sealing possible without grease, contrary to instruction manual • Significant bubble formation	• No leakage problems once cover slip sizing issue addressed • Flow rates up to 60 ml/min
**Major Strength**	Straightforward assembly	Stage adaptor increases flexibility	Chamber fits together well	No leakage, No bubbles
**Major Weakness**	Leakage, no heating apparatus included	Leakage, bubbles	Leakage, bubbles	No heating apparatus included

## Discussion

Cell perfusion chambers provide a tool to probe and elucidate changes in cell physiology in response to mechanical and biochemical stimuli and can be implemented for cytotoxicity and pharmacokinetic studies as well. A given chamber's utility hinges upon its capacity to provide a controlled and known mechanical force that can also emulate aspects of the cell's physiologic environment. Numerous chamber designs have been implemented in a variety of *in vitro *studies; however a recent analysis of chamber performance has shown that several commercial chambers deliver spatially varying and unexpected shear stress distributions [20]. Current commercial chambers have been found to impart mechanical stresses that differ from the target magnitude. Even when the desired stress level is achieved, the area in which the target stress is achieved varies in size and location (between chambers) and is difficult to predict. These shortcomings not only increase the difficulty in making comparisons across studies (e.g. between studies using different chambers, and/or between different experiments using the same chamber) but also provide the potential for misinterpretation of cellular response to flow. Thus, there is a need to improve the current state-of-the-art flow chambers and provide a device to that can accurately impart mechanical stress for in vitro cell studies.

The dual-flow chamber design presented in this study addresses many of the issues associated with previous chambers, and is able to impart a variety of controlled mechanical environments through five gasket designs as well as both single- and dual-flow regimes. Using Gasket I, a constant shear stress is applied over the majority of the chamber area, where 95% of the chamber midplane and 50% of the entire chamber is within 5% of the target stress for both the single and dual-flow setup. Furthermore, if a region of interest is considered where cellular response is likely to be observed, an 8 mm circle at the chamber center, over 92% of the region is within 2.5% of the target stress. This gasket design shows an improvement in both achieving the target stress as well as yielding a predictable location of the desired stimulus. Furthermore, the other gasket designs in this study have specific geometry that allow for precise gradients of shear stress to be applied to a cell monolayer, where the desired gradient can be achieved by observing cellular response at the appropriate distance from the gasket inlet. These stress distributions provide for application of "tunable" mechanical regimes appropriate for modelling a variety of physiological scenarios. In all cases, the imparted shear stress distributions are accurate for both modes of flow (single and dual) as well as standard gasket thicknesses of 250 μm and 500 μm.

As this study provides a design and analysis of a cell perfusion chamber using computational methods, certain limitations can occur. All three-dimensional flow simulations have been checked for accuracy using variations of known analytical solutions, where possible. In addition, results are shown for a target shear stress of 1 dyn/cm^2^, however as the flow regimes are laminar, the predictions shear stresses are scalable to incorporate a wide range of commonly used stress magnitudes (0.1 – 20 dyn/cm^2^).

An understanding of the interplay between cells and their enviroment is critical to elucidating mechanisms of physiological and pathophysiological processes. All life on Earth originated from water and fluids comprise at least part of the environment of every cell in every organism on Earth. Throughout a human's lifespan, every cell in the body is bathed in fluid and is hence subjected to deviatoric shear stresses resulting from fluid flows over apical and/or basal surfaces due to pressure gradients arising from breathing, pumping of the heart, and weight bearing in a gravitational environment. These flows may be superimposed with dilatational tensile and/or compressive stresses that occur when a tissue is deformed during breathing, pumping of the heart, and weight bearing. The relative contribution of dilational and deviatoric stresses varies widely depending on the location of the cell within a given tissue, the location of the tissue within the organism, as well as the time in the lifecycle of the organism. For instance, prior to development of the heart *in utero *(which precedes the development of the circulatory system and the musculoskeletal system), forces associated with cell proliferation, epithelial-mesenchymal transformations, patterning and morphogenesis dominate the mechanical environment of a cell [21,22].

The novel prototype presented here lends itself particularly for cell mechanotransduction studies [26,27] as well as cytotoxicity and pharmacokinetic studies when used to deliver spatiotemporally controlled concentration gradients of molecules to cells seeded within. A new uniaxial strain module is in development, to deliver strains basally in combination with apical flows or concomitant to basal flows. Temporal variation in mechanical stresses and/or molecular concentrations is achieved through specification of flow velocities and/or temporally varying flows and/or strains driven by function generators in combination with programmable syringe pumps. Combined with the gaskets, which provide flow regimes designed to mimic flows typical in lymph vessels, brain, proximal tubules, and blood vessels [28-36], the delivery of spatiotemporally defined molecular gradients to the apical and basal surfaces of cells and tissue slices is an application that is currently being reduced to practice.

## Conclusion

The perfusion flow chamber technology described in this manuscript overcomes several major drawbacks that are currently associated with existing perfusion flow chambers on the market. Firstly, the flow regime in the novel chamber mimics physiologic conditions, which facilitates the development and translation of cell experiments to clinically relevant scenarios. The new design allows for both the quantification and measured control of the shear stress on cells, as opposed to existing designs, which have unknown or variable shear stress. Carefully controlling the stresses on cells is critical in effectively mimicking *in vivo *situations. Overall, the improved perfusion flow chamber provides the needed resolution, standardization and *in vitro *model analogous to *in vivo *conditions to make the step towards greater use in research and the opportunity to enter the diagnostic and therapeutic market.

## Competing interests

The academic institution of the co-authors previously had a non-exclusive licensing agreement with Warner Instruments to produce and market the technology described in this manuscript. To date (26 November 2007), none of the authors has received reimbursements, fees, funding, or salary from Warner Instruments.

## Authors' contributions

MKT developed the modular dual flow chamber concept as well as the concept of defining gasket geometry to affect constant magnitude stress and stress gradients to mimic different physiological situations. EA carried out the computational fluid dynamics simulations and was involved in the design of the novel flow chamber and gasket geometries as well as drafting and revised the manuscript. MKT conceived of and supervised all aspects of the study, as well as drafting and revising the manuscript.

## References

[B1] Albuquerque ML, Flozak AS (2003). Lamellipodial motility in wounded endothelial cells exposed to physiologic flow is associated with different patterns of beta1-integrin and vinculin localization. J Cell Physio.

[B2] Hung CT, Pollack SR, Reilly TM, Brighton CT (1995). Real-time calcium response of cultured bone cells to fluid flow. Clin Orthop.

[B3] Jacobs CR, Yellowley CE, Davis BR, Zhou Z, Cimbala JM, Donahue HJ (1998). Differential effect of steady versus oscillating flow on bone cells. J Biomech.

[B4] Sorkin AM, Dee KC, Knothe Tate ML (2004). "Culture shock" from the bone cell's perspective: emulating physiological conditions for mechanobiological investigations. Am J Physiol Cell Physiol.

[B5] Nauman EA, Satcher RL, Keaveny TM, Halloran BP, Bikle DD (2001). Osteoblasts respond to pulsatile fluid flow with short-term increases in PGE(2) but no change in mineralization. J Appl Physiol.

[B6] Pavalko FM, Gerard RL, Ponik SM, Gallagher PJ, Jin Y, Norvell SM (2003). Fluid shear stress inhibits TNF-alpha-induced apoptosis in osteoblasts: a role for fluid shear stress-induced activation of PI3-kinase and inhibition of caspase-3. J Cell Physiol.

[B7] Butler PJ, Norwich G, Weinbaum S, Chien S (2001). Shear stress induces a time- and position-dependent increase in endothelial cell membrane fluidity. Am J Physiol Cell Physiol.

[B8] Essig M, Friedlander G (2003). Shear-stress-responsive signal transduction mechanisms in renal proximal tubule cells. Curr Opin Nephrol Hypertens.

[B9] Yellowley CE, Jacobs CR, Donahue HJ (1999). Mechanisms contributing to fluid-flow-induced Ca2+ mobilization in articular chondrocytes. J Cell Physiol.

[B10] Archambault JM, Elfervig-Wall MK, Tsuzaki M, Herzog W, Banes AJ (2002). Rabbit tendon cells produce MMP-3 in response to fluid flow without significant calcium transients. J Biomech.

[B11] Cinamon G, Alon R (2003). A real time in vitro assay for studying leukocyte transendothelial migration under physiological flow conditions. J Immunol Methods.

[B12] Forlow SB, McEver RP, Nollert MU (2000). Leukocyte-leukocyte interactions mediated by platelet microparticles under flow. Blood.

[B13] Bilek AM, Dee KC, Gaver DP (2003). Mechanisms of surface-tension-induced epithelial cell damage in a model of pulmonary airway reopening. J Appl Physiol.

[B14] Allen FD, Hung CT, Pollack SR, Brighton CT (2000). Serum modulates the intracellular calcium response of primary cultured bone cells to shear flow. J Biomech.

[B15] Donahue SW, Donahue HJ, Jacobs CR (2003). Osteoblastic cells have refractory periods for fluid-flow-induced intracellular calcium oscillations for short bouts of flow and display multiple low-magnitude oscillations during long-term flow. J Biomech.

[B16] Salih V, Greenwald SE, Chong CF, Coumbe A, Berry CL (1992). The development of an in-vitro perfusion system for studies on cultured cells. Int J Exp Pathol.

[B17] Busscher HJ, van der Mei HC (1995). Use of flow chamber devices and image analysis methods to study microbial adhesion. Methods Enzymol.

[B18] Brown DC, Larson RS (2001). Improvements to parallel plate flow chambers to reduce reagent and cellular requirements. BMC Immunol.

[B19] Chotard-Ghodsnia R, Drochon A, Grebe R (2002). A new flow chamber for the study of shear stress and transmural pressure upon cells adhering to a porous biomaterial. J Biomech Eng.

[B20] Anderson EJ, Falls TD, Sorkin AM, Knothe Tate ML (2006). The imperative for controlled mechanical stresses in unravelling cellular mechanisms of mechotransduction. Biomed Eng Online.

[B21] Anderson EJ, Knothe Tate ML (2007). Design of tissue engineering scaffolds as delivery devices for mechanical and mechanically modulated signals. Tissue Eng.

[B22] McBride SH (2007). Elucidating biophysical cues conducive to targeted multipotent cell differentiation. Master of Science Thesis.

[B23] Anderson EJ, Kaliyamoorthy S, Alexander JID, Knothe Tate ML (2005). Nano-microscale models of periosteocytic flow show differences in stresses imparted to cell body and processes. Ann Biomed Eng.

[B24] Knothe Tate ML (2003). "Whither flows the fluid in bone?" An osteocyte's perspective. J Biomech.

[B25] Knothe Tate ML, Knothe U (2000). An ex vivo model to study transport processes and fluid flow in loaded bone. J Biomech.

[B26] Oberhofer K (2005). Does mechanical stress promote bone cell fate in the murine embryonic mesenchymal stem cell line C3H/10T1/2?. Diploma Thesis.

[B27] Falls TD (2007). Beta-catenin's role in the mechanosensitivity of osteochondroprogenitor cells. Master of Science Thesis.

[B28] DeGrendale HC, Estess P, Picker LJ, Siegelman MH (1996). CD44 and its ligand hyaluronate mediate rolling under physiologic flow: a novel lymphocyte-endothelial cell primary adhesion pathway. J Exp Med.

[B29] Ando J, Tsuboi H, Korenaga R, Takada Y, Toyama-Sorimachi N, Miyasaka M, Kamiya A (1994). Shear stress inhibits adhesion of cultured mouse endothelial cells to lymphocytes by downregulating VCAM-1 expression. Am J Physiol.

[B30] Mairey E, Genovesio A, Donnadieu E, Bernard C, Jaubert F, Pinard E, Seylaz J, Olivo-Marin JC, Nassif X, Dumenil G (2006). Cerebral microcirculation shear stress levels determine neisseria meningitides attachment sites along the blood-brain barrier. J Exp Med.

[B31] Fujiwara T, Akita H, Furukawa K, Ushida T, Mizuguchi H, Harashima H (2006). Impact of convective flow on the cellular uptake and transfection activity of lipoplex and adenovirus. Biol Pharm Bull.

[B32] Yee A, Sakurai Y, Eskin SG, McIntire LV (2006). A validated system for simulating common carotid arterial flow in vitro: alteration of endothelial cell response. Ann Biomed Eng.

[B33] McKinney VZ, Rinker KD, Truskey GA (2006). Normal and shear stresses influence the spatial distribution of intracellular adhesion molecule-1 expression in human umbilical vein endothelial cells exposed to sudden expansion flow. J Biomech.

[B34] DePaola N, Gimbrone MA, Davie PF, Dewey CFJ (1992). Vascular endothelium responds to fluid shear stress gradients. Arterioscler Thromb.

[B35] DePaola N, Davies PF, Pritchard WF, Florez L, Harbeck N, Polacek DC (1999). Spatial and temporal regulation of gap junction connexin43 in vascular endothelial cells exposed to controlled disturbed flows in vitro. Proc Natl Acad Sci USA.

[B36] Nagel T, Resnick N, Dewey CF, Gimbrone MA (1999). Vascular endothelial cells respond to spatial gradients in fluid shear stress by enhanced activation of transcription factors. Arterioscler Thromb Vasc Biol.

[B37] ProFlow single and dual layer flow cells. http://www.mechbio.org/proflow.

